# Effect of Femoral Head Radial Clearance on Acetabular Cartilage Degradation in Hip Hemiarthroplasty: An In Vitro Anatomical Simulator Study

**DOI:** 10.3390/bioengineering13070783

**Published:** 2026-07-07

**Authors:** Roberto Leonardo Diaz, David Jimenez-Cruz, Tim N. Board, Sophie Williams

**Affiliations:** 1 Institute of Medical and Biomedical Engineering, School of Mechanical Engineering, University of Leeds, Leeds LS2 9JT, UK; davidjimc@gmail.com (D.J.-C.); s.d.williams@leeds.ac.uk (S.W.); 2 Wrightington Hospital, Wigan and Leigh NHS Foundation Trust, Lancashire WN6 9EP, UK; tim@timboard.co.uk

**Keywords:** hip hemiarthroplasty, cartilage degradation, anatomical hip simulator, tribology, cartilage wear

## Abstract

Over 30,000 hip hemiarthroplasty (HA) operations are performed every year across England and Wales to treat fractured necks of the femur. HA reduces surgical and recovery time with lower complication rates; however, it may cause acetabular deterioration, which can lead to revision surgery and possible conversion to total hip arthroplasty (THA). This study assessed hip hemiarthroplasty under gait-representative loading in an anatomical hip simulator. Paired natural acetabula were tested against a CoCr femoral head with radial clearance (RC) of −0.75 mm (head larger than natural) and positive RCs of <0.6 mm (small), 2 mm–4 mm (large), and >4 mm (extra-large). Cartilage surface deterioration was quantified via photogrammetry. Cartilage surface changes were observed in all hemiarthroplasty groups, while no changes were observed in the control group. No statistically significant between-groups in the affected area were detected (Kruskal–Wallis, *p* > 0.29). The negative RC group showed statistically significant progressive worsening over time (Friedman: (χ^2^(2) = 8.00, *p* = 0.018). Groups differed in damage onset, location, intensity, and presence of delamination. Samples with negative RC (oversized head) produce earlier and progressive cartilage changes. The results highlight the importance of carefully measuring the native head diameter and choosing a femoral head size when performing HA.

## 1. Introduction

Hemiarthroplasty of the hip (HA), where an artificial femoral head on a stem articulates with the natural acetabulum, remains the most frequently performed operation in managing a fractured neck of the femur (National Hip Fracture Database 2019) [1]. Across England and Wales over 30,000 HAs are carried out every year [2]. The prevalence of HA procedures may be due to shorter surgical times, ease of operation, reduced recovery time, closer preservation of the native hip compared to a total hip replacement (THA), and lower complication rates. A metanalysis has shown no difference in reoperation rate and a clinically insignificant increase in function and quality of life with THA compared to hemiarthroplasty [3,4,5].

The main disadvantage of hip hemiarthroplasty is subsequent acetabular cartilage deterioration, which can lead to pain and the requirement for revision surgery and conversion to THA. The continued use of HA devices demonstrates that there is a need to investigate their efficacy pre-clinically and parameters which may reduce cartilage erosion caused by articulating an artificial femoral head against the natural acetabulum. However, to date, reports of methodologies for such in vitro pre-clinical testing have been limited. Inaccurate femoral head sizing is a clinically significant and common problem: standard digital templating fails to predict the correct head size to within 1.4–1.7 mm in more than 50% of cases [6,7], and conventional intraoperative visual estimation causes substantial leg-length discrepancy in approximately 40% of patients [8,9]. These systematic sizing errors directly accelerate acetabular erosion and increase long-term revision risk [8].

A previous in vitro investigation examined acetabulum cartilage wear in HA using a pendulum friction simulator with hip motion limited to flexion and extension [10]. Lizhang et al. [11] paired porcine acetabula to metal femoral heads with different diameters to allow hip joint clearance. Their findings reported more significant cartilage deformation in joints with small or extra-large clearance. Although these results are relevant, the simulation was limited to unidirectional motion.

The aim of this study was to assess the effects of fit discrepancy between the natural acetabulum and replaced femoral head in an experimental simulation of hemiarthroplasty that applied loading and motion cycles mimicking gait. The resultant degradation of cartilage was recorded, and the changing contact mechanics were assessed.

## 2. Materials and Methods

### 2.1. Overview

Hip hemiarthroplasty was investigated using porcine acetabula paired with metal femoral heads. The tissue was dissected, and the acetabula was paired with a metal femoral head and mounted in an experimental hip simulator. Clinically relevant cyclical loads and motions were applied for eight hours. Changes in the acetabular cartilage surfaces were recorded with photogrammetry and contact conditions in the hemiarthroplasty were considered using Hertzian theory. The methodology is described in detail in the following sections.

### 2.2. Tissue Preparation

Right hind porcine legs were obtained at the local abattoir (John Penny & Sons, Leeds, UK) within 24–48 h of slaughter. Animals were approximately six months of age at slaughter, corresponding to commercially mature but skeletal immature pigs). The study included twenty hip joints from pigs of similar weight (mean weight 84 kg). Specimens were dissected within 3 h of collection and stored at 4 °C. To maintain hydration during the interval prior to testing, samples were placed in sealed bags containing phosphate-buffered saline (PBS)-soaked gauze. Musculature and soft tissue were removed from the specimens, leaving the joint capsule intact; then the joint was carefully disarticulated by incising and then removing the capsule, dislocating the femoral head, and removing the ligament *teres*. The labrum and cartilage were preserved intact and kept hydrated by spraying with phosphate-buffered saline (PBS) throughout the sample preparation process.

The diameter of the femoral head was measured using scaled circular templates. The head was inserted into the diameter of the gauge, keeping the epiphyseal line parallel to the template edge. The size of the femoral head was registered as the smallest diameter where the head freely fitted into the template. This measurement was later used to determine the size of the metal head to be paired with the corresponding acetabulum in the HA experimental testing. The circular template provides measurements at 1.0 mm diameter increments, yielding an inherent sizing uncertainty of ±0.5 mm in diameter (±0.25 mm in radius). This propagates as a static ±0.25 mm systematic uncertainty in the calculated baseline radial clearance, introduced solely during the initial component sizing phase and not affecting further stages of the simulation testing.

### 2.3. Hemiarthroplasty Joint Models

Hemiarthroplasty test samples comprised paired natural acetabula with CoCr femoral heads. The clearance between the acetabulum and femoral head varied between test samples. The radial clearance (RC) was calculated as the radius of the natural femoral head minus the radius of the artificial head. A positive RC is a head smaller than a natural femoral head and the acetabulum (greater incongruence), while a negative RC corresponds to a head larger than the natural femoral head. By referencing the measured native femoral head diameter of each specimen, CoCr heads with RCs of <0.6 mm (small RC), between 2 mm and 4 mm (large RC), and >4 mm (extra-large RC) were tested. A further sample with negative RC of −0.75 mm (CoCr head larger than the natural head) was tested. A control group (*n* = 4) consisting of natural joints was also assessed (Table 1).

**Figure 1:**
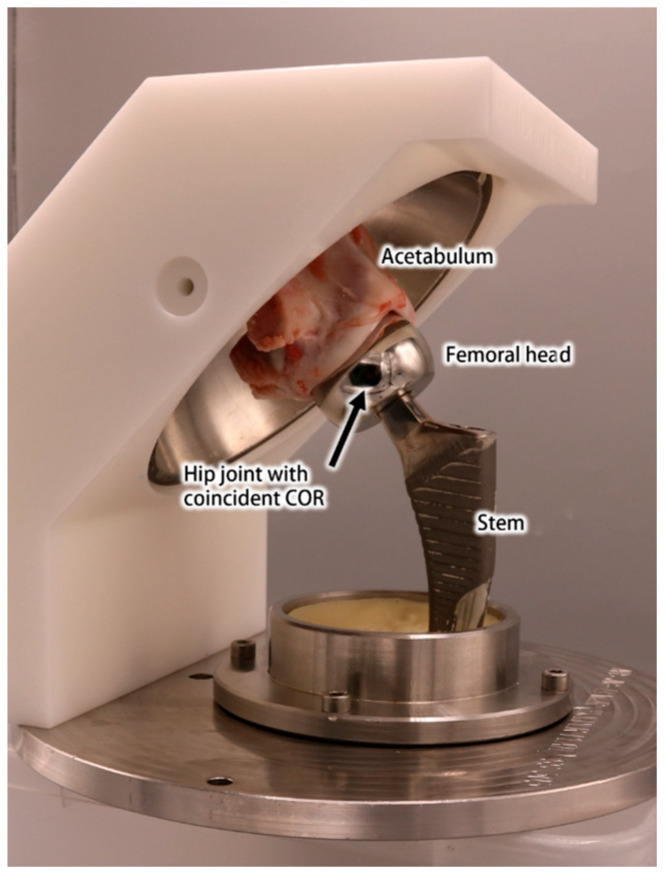
Hemiarthroplasty sample preparation. Assembly of the sample uses custom fixtures to align the centre of rotation of the acetabulum, femoral head and simulator. The acetabulum is anatomically oriented using the *TAL* as a reference. Tissue is held between a femoral prop and posterior screws during cementation.

The natural acetabulum and femoral stem were individually cemented (PMMA, polymethyl methacrylate) into metallic fixtures and tested in an anatomical hip simulator. The acetabula were oriented anatomically using the transversal acetabular ligament (TAL) and labrum rim. Custom fixtures were used to align the centre of rotation (COR) of the joint with the COR of the simulator, Figure 1. The natural acetabula were subsequently cemented to ensure anatomical alignment in the simulator, with the heads mounted on femoral stems.

### 2.4. Anatomical Hip Simulator

**Figure 2:**
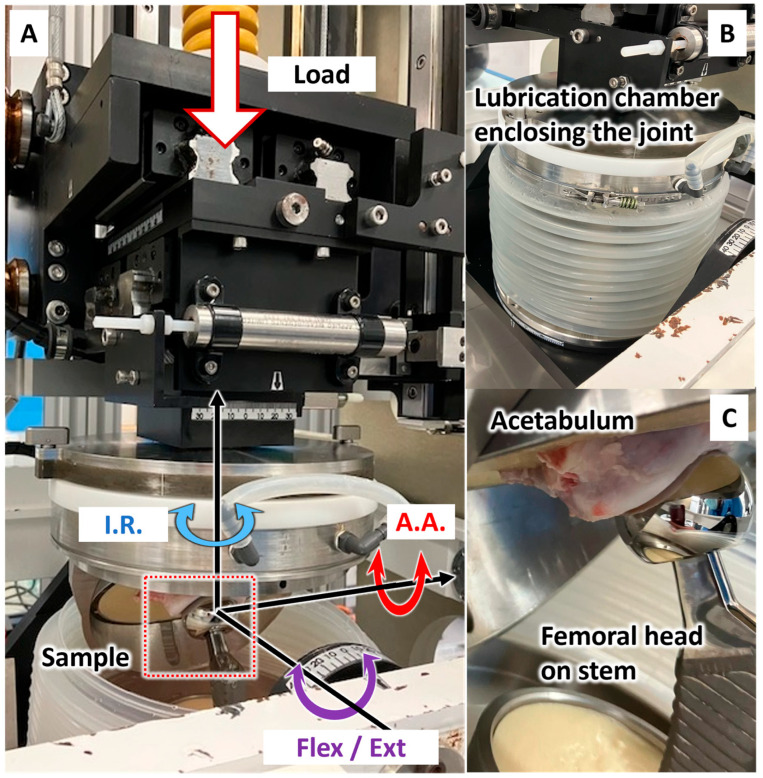
Experimental configuration in the anatomical hip simulator (AHS). (**A**) The acetabular component is fastened to a carriage with one degree of freedom to apply force. The femoral component is secured to a pendulum carriage that can concurrently rotate around the joint’s centre of rotation. A schematic illustration at the COR illustrating the axis orientation for flexion/extension (Flex/Ext, purple arrow), abduction-adduction (A.A., red arrow), and internal-external rotation (I.R., blue arrow). The red square highlights the location of the joint, which is shown in detail in panel C (**B**) Test rig with lubrication chamber in place, containing PBS as a lubricant. (**C**) Zoom to the hemiarthroplasty model.

The experimental test was conducted in vitro using an electromechanical anatomical hip simulator (ProSim, Stockport, Manchester, UK). The simulator reproduced representative human hip motion, by applying flexion-extension, adduction-abduction and internal-external rotation to the femoral head, while applying an axial load to the acetabulum (Figure 2). Samples were enclosed in a silicone chamber filled with phosphate-buffered saline (PBS) solution throughout testing, providing a physiologically lubrication environment. The input motion profile consisted of a simplified twin peak gait cycle adapted from the ISO 14242 standard “Wear of hip-joint prostheses”. Because porcine tissue was used in this study, the profile was adapted to account the smaller range of motion and the reaction forces experienced in these animals [12,13,14,15]. This profile included a peak load of 900 N, 20° to −20° flexion-extension, −8.8° to 4.8° of abduction-adduction, and 2° to −10° of internal-external rotation [16]. The medial-lateral axis was free to allow slight adjustment of the hip joint centre of rotation. The test duration was 28,800 cycles (8 h) at 1 Hz, with photogrammetry performed at 1-h intervals.

### 2.5. Cartilage Degradation Assessment Methods

Photogrammetry was used to document and quantify macroscopic changes to the acetabular articular surface at hourly intervals (every 3600 cycles). Photographs were taken using a Canon 750D DSLR camera equipped with wide-angle lens (EF-S 28–70 mm F3.5–5.6) and macro lens (EF 100 mm f2.8 USM), within a lightbox to maintain consistent camera settings and lighting conditions throughout testing. Upon removal from the simulator at each interval, the cartilage was allowed to recover for approximately ten minutes before photography. Alterations in the articular surfaces were documented, including modifications in colour, blushing, cartilage delamination, abrasions, indentations, and preservation of the labrum. Surface irregularities were defined as any visible disruption to the smooth articular surface, including pitting, fibrillation, or localised surface depression detectable under standardised photographic conditions. Coloured areas on the acetabulum due to application of load were quantified on the photographs after one, four, and eight hours of testing. The areas were quantified using image processing software (ImageJ 1.52a, Bethesda, MD, USA) by using image thresholding in order to identify contrast edges. The degraded areas were reported as a ratio to the total cartilage area that was visible in the image (Degraded/Pre-test Ratio, D/PTR). RGB colour values were recorded to quantify the intensity of the blushing. This approach was taken to reduce inaccuracies associated with anatomical singularities of the specimen and the estimation of areas projected in a 2D image. The D/PTR metric uses each specimen’s own pre-test image as a reference baseline, reducing the influence of inter-specimen colour variation. Deformation of the cartilage surface was assessed visually immediately after removal form the simulator and again after a recovery period of 14 h of hydration in PBS.

The macroscopic damage observations are associated with the tissue degradation ranking show in Table 2. The ranking was adapted from established grading systems (Outerbridge, ICRS, and OARSI) [14] and the chondro-labral visual damage grading system [17] validated against histopathology in comparable in vitro natural hip simulation studies. Each level integrates the macroscopic surface features observable by photogrammetry (surface discolouration, blushing, deformation, and delamination) with their corresponding structural significance.

### 2.6. Theoretical Assessment of Contact Conditions in Hemi-Arthroplasty Joints

**Figure 3:**
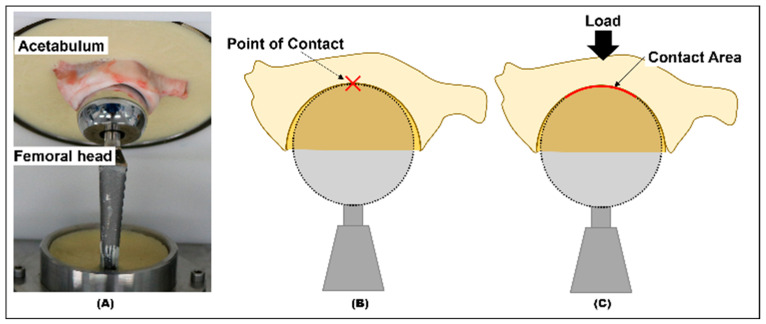
Hertzian contact for hemiarthroplasty model. (**A**) Experimental HA model, (**B**) Hertzian spherical contact assumes one point of contact between two interacting spherical surfaces before a load is applied. (**C**) When the load is applied, the bodies experience elastic deformation, producing a contact area, pressure, and stress.

The contact mechanics of the hip joint, in which a semi-spherical cavity of the acetabulum comes into contact with a spherical femoral head, can be examined as a conformal contact system. Assessing the contact mechanics provides an insight into the contact pressure, and contact area generated on the bearing surfaces of the joint. In the hemiarthroplasty model, the convex and concave surfaces of the joint initially make contact, and after experiencing an external force, the contact area increases (Figure 3). This contact causes the acetabular surface to undergo elastic deformation, depending on the material properties, lubrication, load, and clearance. For this study, the contact area and contact pressure were calculated using adapted formulae from tribology studies, Equations (1)–(4) [23,24].

(1) $$\mathrm{Contact}\mathrm{radius}\;\;a=\sqrt[{3}]{\left(\frac{3FR_{e}}{2E^{*}}\right)}$$

(2) $$\mathrm{Maximum}\mathrm{pressure}\;\;P_{max}=\frac{3F}{2\pi \;a^{2}}$$

(3) $$\mathrm{Equivalent}\mathrm{radius}\;R_{e}=\frac{R_{FH}(R_{FH}+C)}{C}$$

(4) $$\mathrm{Equivalent}\mathrm{elastic}\mathrm{modulus}\;\;E^{*}=2{\left(\frac{1-\nu_{FH}^{2}}{E_{FH}}+\frac{1-\nu_{AC}^{2}}{E_{AC}}\right)}^{-1}$$

where *F* corresponds to the maximum force applied (900 N), and *C* corresponds to the radial clearance increment in the joint. The mechanical properties for the CoCr of the femoral head are *ν_FH_* = 0.3 and *E_FH_* = 220,000 MPa [25]. The mechanical properties of the acetabular cartilage are *ν_AC_* = 0.08 and *E_AC_* = 4.5 MPa [26,27].

Although articular cartilage is a viscoelastic, biphasic, and anisotropic tissue, Hertzian theory is applied in this study as an analytically tractable first order estimate for the purposes of comparative analysis across clearance groups. This approach is most valid at the onset of elastic deformation, which is the loading regime most relevant to acute damage initiation. Recent work by Peñaherrera-Carrillo et al. [28], explicitly demonstrates that Hertzian-based models can reliably identify and rank clinically meaningful contact pressure differences across joint conditions, noting that the geometry changes had greater influence on contact mechanics than stiffness or load changes. These results support the validity of Hertzian theory as a comparative tool even when individual absolute values carry acknowledged uncertainty.

### 2.7. Statistical Analysis

All statistical analysis was performed using MATLAB (R2023a, MathWorks, Natick, MA, USA). Data are reported as median (interquartile range (IQR)) and mean ± standard deviation (SD). Given the small sample sizes (*n* = 4 per group), non-parametric tests were used throughout. Between-group differences in D/PTR were assessed at timepoints (1 h, 4 h, and 8 h) using the Kruskal–Wallis test. Where a significant result was obtained, Dunn’s post-hoc test with Bonferroni correction was applied for pairwise comparisons. Within-group changes in D/PTR overtime were assessed using the Friedman test and the non-parametric equivalent of repeated-measures ANOVA, and the same specimen was measured at multiple timepoints. Where a significant Friedman result was obtained, Dunn’s post-hoc test with Bonferroni correction was applied to pairwise timepoint comparisons (1 h vs. 4 h, 1 h vs. 8 h, and 4 h vs. 8 h). A *p*-value of less than 0.05 was considered statistically significant.

## 3. Results

A total of twenty-four porcine hip joints were tested in the anatomical hip simulator; twenty joints were included in the study, while four were excluded due to technical issues resulting in premature tissue degradation or failure. Photogrammetry was used to evaluate the deterioration of cartilage, and labrum of successful test, by comparing photos captured during testing. The dataset associated with this study (photographic record) is openly available from the University of Leeds repository [29].

### 3.1. Cartilage Degradation

Porcine hips were subjected to an eight-hour test in the AHS. No alterations were observed in the macroscopic assessment of the cartilage surface in the control group (natural joint). The cartilage was free of surface irregularities, scratches, or deformation; and there were no tears or separation observed in the labrum.

**Figure 4:**
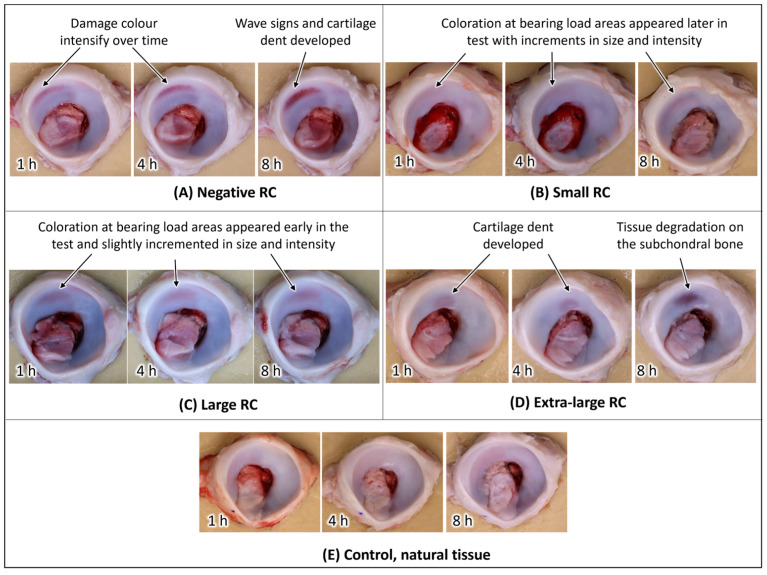
Cartilage blushing appeared at the posterior-superior area. (**A**) Negative radial clearance (RC) group, (**B**) Small RC group, (**C**) Large RC group. (**D**) Extra-large RC group. (**E**) Control group with natural tissue showed no changes.

In all hemiarthroplasty groups, cartilage changes were noted, and these are summarised in Figure 4.

In the negative RC group (RC = −0.75), cartilage surface deformations and subchondral blushing were visible from the second hour of testing at the posterior-superior edge of the acetabular rim. The median D/PTR after one hour was 5.0% (mean 5.6% ± 2.7%), rising to 10.4% (mean 10.5% ± 2.0%) after eight hours. The Friedman test indicated that this progressive increase over time was statistically significant (χ^2^ (2) = 8.00, *p* = 0.018), confirming that cartilage surface change in this group worsened continuously throughout the test. Cartilage delamination became apparent after two hours.

In the small RC group (RC < 0.6 mm), acetabular blushing began to appear at the posterior superior edge after four hours of testing. The median D/PTR after one hour was 2.2% (mean 2.5% ± 2.9%), increasing to 9.7% (mean 9.4% ± 5.3%) after eight hours. The within-group change over time was not statistically significant (Friedman: *p* = 0.085), and the wide IQR at eight hours (4.9–14.0%) reflects considerable variability between specimens. The intensity of surface discolouration was comparatively less pronounced than in other groups. All samples showed no discernible deformations, delamination, debonding, or damage to the labrum, consistent with the lower contact stress associated with the least incongruent group.

The large RC group (2 mm < RC < 4 mm) exhibited acetabular blushing in the posterior-superior region, at the radial centre of the acetabular annulus. This was visible after one hour of testing, with a median D/PTR of 7.0% (mean 7.3% ± 4.0%), increasing to 9.7% (mean 9.7% ± 3.4%) after eight hours. The within-group progression over time was not statistically significant (Friedman: *p* = 0.105). The intensity of the lesion was apparent from the first hour and did not increase substantially throughout the test, suggesting that the initial contact conditions drove the majority of the observable change. All specimens exhibited no visible or permanent deformations, delamination, debonding, or damage to the labrum.

The extra-large RC group (4 mm < RC) presented cartilage surface deformations and subchondral blushing within the first hour of testing, in the posterior-superior region proximal to the inner edge of the acetabular fossa. The median D/PTR after one hour was 7.4% (mean 7.2% ± 4.2%), rising to 11.5% (mean 10.9% ± 4.3%) after eight hours. The within-group change over time was not statistically significant (Friedman: *p* = 0.368), though the lesion intensity increased and was pronounced from the first hour. Specimens developed deformations on the cartilage, with some displaying delamination but no evidence of debonding. There was no presence of labrum damage. This group showed the greatest positive incongruence among the hemiarthroplasty groups.

**Figure 5:**
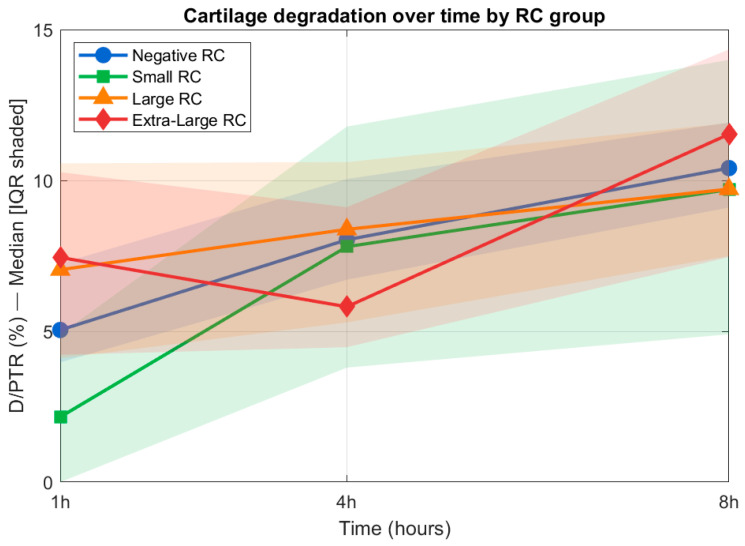
Degraded-to-Pre-test Ratio (D/PTR, %) over time for each radial clearance group. Lines represent group medians; shaded regions represent the interquartile range (IQR). The Negative RC group (oversized femoral head) demonstrated a statistically significant progressive increase in D/PTR from 1 h to 8 h (Friedman test: χ^2^(2) = 8.00, *p* = 0.018). No other group showed a statistically significant change over time. The transient decrease observed in the Extra-Large RC group at 4 h reflects specimen variability within this group (*n* = 4).

D/PTR values across all groups were compared at each timepoint using the Kruskal–Wallis test. Not statistically significant between-group differences were detected at one hour (H(3) = 3.73, *p* = 0.292, four hours (H(3) = 0.64, *p* = 0.888), or eight hours (H(3) = 0.41, *p* = 0.937). At eight hours, median (IQR) D/PTR values were 10.4% (2.8) for Negative RC, 9.7% (9.1%) for Small RC, 9.7% (4.4%) for Large RC, and 11.5% (6.9%) for Extra Large RC. The absence of significant between-group differences in total affected area reflects the fact that D/PTR captures the extent of surface discolouration rather than its severity, depth, or morphological character. The groups were distinguished not by the total area of change but by the timing of onset, lesion intensity, and presence of delamination and permanent deformation. Figure 5: Effect sizes for the Kruskal–Wallis tests were small at all timepoints (1 h: ε^2^ = 0.24; 4 h: ε^2^ = 0.04; 8 h: ε^2^ = 0.03), consistent with negligible between-group differences on the D/PTR metric at this sample size. For the Friedman within-group test in the Negative RC group (*p* = 0.018), Kendall’s W = χ^2^/[*n*(k − 1)] = 8.00/(4 × 2) = 1.00, reflecting a monotonic increase in damage rank across all four specimens at each timepoint, a pattern not observed in any other group.

**Figure 6:**
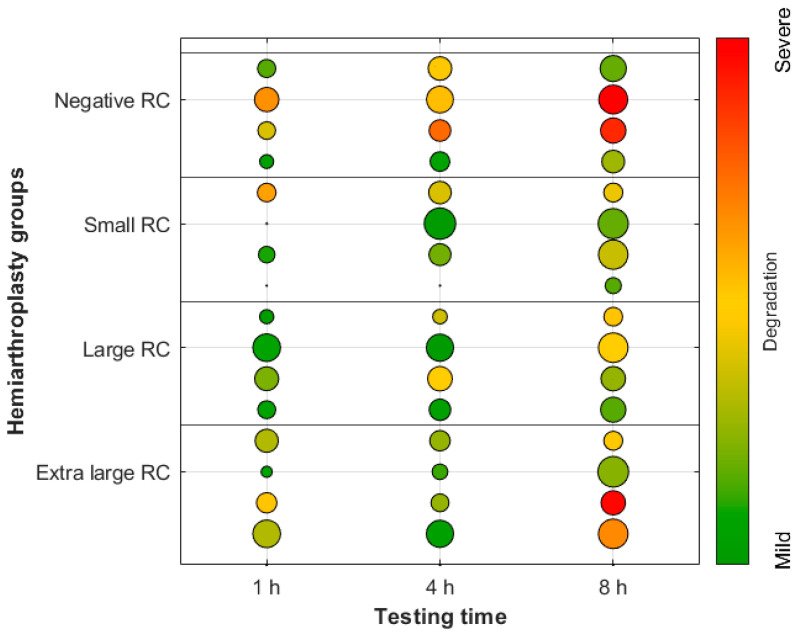
Progress of tissue degradation throughout the test. Individual samples (*n* = 4) are presented in each hemiarthroplasty test group with a different radial clearance (RC), and the progression of damage through the test is shown. The marker size represents the degraded/pre-test ratio areas on the acetabulum (D/PTR) and the intensity of colour indicates the severity of degradation observed. The gradient ranges from green (mild) to red (severe). The native/control group was not included since it did not show signs of tissue degradation.

The tissue degradation progression for each study group throughout the test is presented in Figure 6.

**Figure 7:**
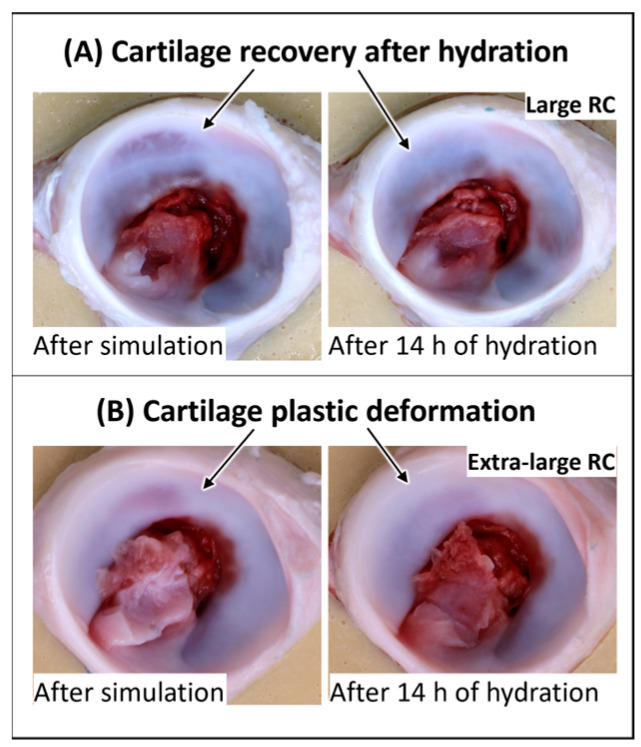
Cartilage deformation during test. Images were taken after four hours of testing and after a fourteen-hour cycle of hydration with PBS in a sealed bag. Subchondral colour and deformation reappeared after resuming the test.

Testing revealed elastic deformation of the cartilage. Deformation of the articular surface was visible immediately after removing the sample from the anatomical simulator and disappeared during the photogrammetry process (Figure 7). As the test continued, tissue damage in the form of a bruise occurred in the same area as the deformations. Some deformations progressed to plastic deformation, even after undergoing a prolonged period of hydration recovery. The control group had no observable differences between the pre-and post-tests. Although the effect differed among the hemiarthroplasty groups, the majority of the samples in the extra-large group experienced plastic deformation.

### 3.2. Hertzian Contact Area for Hemiarthroplasty

The Hertzian contact pressure was calculated based on the increment of the radial clearance for each group (Table 3). The contact pressure was approximately 2.0 MPa for the natural tissue (control) group, 3.3 MPa for the negative RC and the small RC groups, and 3.5 MPa for the large and extra-large RC groups. In terms of contact, the contact area estimated for the control group was up to 50% larger than any samples in the hemiarthroplasty group. A Spearman rank correlation between group mean Hertzian contact pressure and group mean D/PTR at 8 h yielded a positive directional association consistent with the proposed pressure–damage relationship; however, with four data points, this did not reach statistical significance and should be interpreted with caution. Notably, the peak contact pressure range across hemiarthroplasty groups is narrow (3.30–3.49 MPa); the more mechanistically informative distinctions between groups lie in contact area and depth of maximum shear stress, both of which scale more substantially with radial clearance.

## 4. Discussion

This study assessed hip hemiarthroplasty under clinically relevant mechanical conditions to investigate cartilage degradation in relation to femoral head clearance. The use of hemiarthroplasty in the treatment of femoral neck fractures has shown advantages such as lower blood loss, shorter duration of the surgical procedure and lower risk of hip dislocation compared to a total hip arthroplasty; [3,5,30,31], however postoperative complications including progressive wear of the retained acetabular cartilage has been under scrutiny. Our in vitro methodology allowed relevant loading and motion conditions to be applied to HA joints with different clearances to investigate its effect on cartilage degradation patterns in a controlled test.

This study showed damage to the acetabular articular cartilage when subjected to an anatomical loading profile against a metal femoral head. The damage was macroscopic, with different location patterns observed for each clearance group (Figure 6). No abrasive wear was observed on the articular surface; in all cases, the damage appeared as blushing in a subsurface region.

This effect is comparable with the findings on mechanical properties in articular cartilage described by other authors [18,22,32]. Oungoulian et al. [22] conducted friction tests of common implant materials (CoCr alloys, 316SS, and borosilicate glass) against bovine cartilage disks. They reported minimal abrasive damage to the contact surface between the artificial and the natural material but also described the development of a darker narrow band at a subsurface level. Their histological analyses showed that the darker band was the initiation of cartilage delamination from the bone. Further investigation with microscopy demonstrated that the delamination occurred in the middle zone of the articular layer. Similarly, Buckley et al. [18] mapped the shear profile across the total thickness of the articular layer and found that the layer just beneath the superficial zone has the lowest shear modulus; the most significant amount of shear strain occurs in this region, allowing the development of delamination.

Reproducing anatomical motion and loading conditions using the hip simulator generated damage at the acetabulum’s posterior-superior region but at a different radial distance from the acetabular fossa in the different clearance groups. These findings are notable because we were able to see how the damage distributes on the acetabular surface, unlike previous studies investigating the mechanical properties of the cartilage surface using pendulum simulators [11,33] or pin-on-disk studies [18,22,32]. Lizhang et al. [11] measured cartilage deformation using contact profilometry in HA models with different clearance fitting between the acetabula and metal femoral heads. Unlike our results, the damage to the cartilage surface showed semi-circular grooves distributed radially from the fossa, development of the pendular motion was limited to one axis. In a similar pendulum simulator, Groves et al. [10] tested a HA model using matching-size CoCr femoral heads to investigate the tribological performance of HA. Comparable to our results, they reported changes in the articular cartilage in the form of significant discolouration, minor superficial scratches, and deep chondral lesions.

From this study, we found different levels of damage for each clearance group. The discolouration increased every hour in the large and extra-large clearance groups, whereas no significant changes were noted after the first hour in the small clearance group. The reduced degradation in the small clearance group is postulated to be due to lower contact stress. These findings are supported by our calculation using a Hertzian contact model. The contact stress values estimated were similar to those reported in previous studies in porcine models [27,34]. The Hertzian analysis indicates that contact pressure increases markedly with greater incongruence (larger RC), while the contact area simultaneously decreases, a consequence of reduced conformity between the bearing surfaces. This elevated, concentrated contact stress at greater clearances may explain the more widespread and more rapidly progressing blushing and delamination observed in the large and extra-large RC groups. Conversely, the small RC group, with near-native conformity, achieved a broader contact area and lower peak pressure, consistent with its lesser cartilage disruption. A noteworthy observation derived from the Hertzian contact model is that the depth of the maximum shear stress also increases as the contact stress rises. The depth of maximum shear stress, which scales directly with contact radius, increases substantially with radial clearance. At smaller clearances, shear stress remains superficial and the tissue accommodates loading without subsurface failure; at larger clearances, shear stress penetrates deeper into the middle zone, where cartilage has its lowest shear modulus [18], promoting delamination. The contact area varies more substantially across clearance groups than peak contact pressure, and represents the more discriminating mechanical parameter for explaining the observed damage patterns. This effect may explain the fact at lower contact stress, the shear is likely to be mitigated at the cartilage, but at greater values, the shear stress penetrates the subsurface and causes cartilage delamination.

The use of an oversized femoral head (negative RC, approximately −0.75 mm) resulted in the earliest cartilage changes, appearing within two hours. This finding has direct clinical relevance: follow-up studies of patients with HA have reported cartilage deteriorations attributable to overload caused by leg lengthening and the use of femoral components larger than the native head [35]. Our findings support the importance of accurate native femoral head sizing at the time of surgery and caution against implantation of oversized components.

## 5. Limitations

This study has some limitations. This study has only assessed the effects of mechanical degradation of the cartilage; however, acetabular erosion is multifactorial, and the biological and chemical factors were not considered. Separating the impact of natural tissue decay and degradation due to bearing stress on the change of colour of the sample during the test was a significant challenge. In order to mitigate this impact, the colour intensity of the regions of interest was compared to the colour of the non-bearing regions within the same image. No formal inter-rater reliability assessment was performed, which is acknowledged as a limitation; however, D/PTR was used as a comparative indicator of damage extent rather than a precision clinical measurement tool, and the interferences trends within groups are unlike to be affected by minor measurement variability with the standardized protocol.

In vivo animal studies have found evidence of advanced cartilage damage extending to subchondral plate thickness and changes to the porosity of the trabecular bone, which may be linked to cell response [36]. Histological analysis was not performed in the current study. Histology would be required to confirm the nature of the subsurface changes observed; for example, to determine whether proteoglycan loss, collagen disruption, or chondrocyte damage occurred, which are hallmarks of true cartilage degradation. This is acknowledged as a significant limitation, and future work should incorporate histological staining to validate macroscopic observations. To strength the confidence in the macroscopic findings, a tissue degradation level was presented in Table 2. This table integrates stablished grading systems (Outerbridge, ICRS, and OARSI) and macroscopic features observed during the simulation, such as blushing and surface striations that have been previously mapped to changes in the structural damage on the tissue [14,17]. Additional evidence for genuine structural damage includes: macroscopically visible delamination in the negative and extra-large RC groups, a well-characterised shear fatigue failure mode initiating at the sub-superficial/middle zone [18,22]; persistent cartilage deformation in the extra-large RC group following 14 h of PBS hydration recovery, indicating irreversible plastic change; and the statistically significant, monotonic increase in D/PTR over time in the negative RC group (Friedman test, *p* = 0.018), inconsistent with transient or reversible artefacts.

The use of porcine acetabula is a recognised limitation of this study. The porcine specimens used were sourced from the food chain, from animals of approximately six months of age, corresponding to commercially mature but skeletal immature animals. Cartilage from immature animals has different mechanical properties from mature human articular cartilage, including lower stiffness and potentially greater susceptibility to fatigue damage. This may partly explain the extent of cartilage changes observed after only 8 h of testing, and the findings may not be directly translatable to the human clinic scenario. However, this study compares relative differences in onset, severity, morphology, and damage progression using the same tissue type under identical loading conditions, thus the results maintain its mechanical validity as they do not depend by tissue type. The clinical implications are therefore that the absolute magnitude of damage observed in our protocol will not directly replicate what would occur in a human joint, but the relative differences between radial clearance groups, and the biological and mechanical mechanisms of damage, are expected to remain directionally consistent. The trends of damage progression reported in this pilot study should be investigated in future research using human hips. Human articular cartilage from older adults, the population that most commonly requires HA, would have different wear characteristics. Future work should aim to validate findings with human or more mature tissue.

The test duration of 28,800 cycles at 1 Hz (8 h) was selected to capture damage initiation and early progression within a practical laboratory timeframe. Device-measured data indicate that a moderately active patient recovering from hip arthroplasty accumulates fewer than 6000 steps per day [37,38,39], meaning 28,800 cycles correspond approximately to 4.8 days of light walking. The continuous loading protocol does not replicate the intermittent rest periods that allow viscoelastic recovery and fluid redistribution in vivo; the absence of rest periods accelerates biphasic cartilage consolidation and mechanical stress. These results therefore reflect a compressed, acute to sub-acute loading response, damage initiation and early propagation, rather than chronic long-term wear, and should be interpreted accordingly. We considered rounded spheres shapes for the acetabulum in our Hertzian contact analysis for calculation purposes; however the actual acetabular surface is more complex. The application of Hertzian theory to conformal contacts introduces know limitations [40], and the contact pressure values under the loading conditions and clearances reported in this study should be treated as group comparative estimates rather than precise absolute values. The time-dependent poroelastic behaviour of cartilage under sustained or cyclic loading falls outside the scope of this framework [41,42]. Biphasic, fibril-reinforced, or finite element modelling is identified as a priority for future work. Future work may consider excessive pressure on the cartilage and oversizing of the femoral head for the acetabulum.

The statistical power of the between-group comparison was limited by the small sample size of *n* = 4 per group; however, the study is powered to detect trends and establish feasibility as a pilot investigation. A retrospective power analysis indicates that approximately 20–25 specimens per group would be required to achieve 80% power at α = 0.05 for between-group comparisons using D/PTR only. This study should be considered exploratory in nature; future work with a larger cohort is needed to confirm these findings.

## 6. Conclusions

In conclusion, this study highlights the significant impact of clearance variations in hip hemiarthroplasty on acetabular cartilage degradation under simulated anatomically relevant loading conditions for the first time. The findings indicate that larger clearances result in increased damage to the cartilage, characterised by discolouration and subsurface delamination, while smaller clearances exhibit reduced degradation due to lower contact stress. This highlights the importance to surgeons of carefully measuring the native head diameter and choosing a head size just smaller than the native head whilst performing HA. Although the study primarily focuses on mechanical factors, it acknowledges the multifactorial nature of acetabular erosion, suggesting that future research should incorporate biological and chemical influences.

## Figures and Tables

**Table 1:** Radial clearance (RC) defined as the radial difference between the measured native femoral head and CoCr head used in testing, by group (*n* = 4). RC in the control group was 0 mm.

Group	Mean Average RC [mm] (SD)
Negative	−0.75 (0.28)
Small	0.19 (0.40)
Large	1.88 (0.11)
Extra large	3.38 (0.45)

**Table 2:** Tissue degradation level for macroscopic assessment of acetabular cartilage.

TD Level	Macroscopic Feature	Structural Significance	Correspondence to Stablished Scales
Undamaged	Smooth, unaltered articular surface. No discolouration, blushing, deformation, or labral change.	No structural change. Reversible elastic deformation only.	Outerbridge Grade 0; ICRS Grade 0.
Mild surface change	Localised mild blushing or discolouration. Low D/PTR (<5%). No deformation or labral involvement.	Subsurface softening and swelling; early mid-zone collagen disruption consistent with fatigue initiation.	Outerbridge Grade I; ICRS Grade IA.
Moderate surface change	Widespread or progressive blushing. Moderate D/PTR (5–12%). Elastic deformation visible on removal but recovers after hydration. No permanent deformation.	Advancing mid-zone fatigue damage with probable collagen cross-link disruption. Superficial lamina splendens largely intact. Consistent with OARSI Grade I–II surface change.	Outerbridge Grade I–II; ICRS Grade IB–II.
Marked degradation	Pronounced progressive blushing with high D/PTR (>10%) and statistically significant worsening over time. Early onset (within first two hours). No permanent deformation or visible delamination.	Substantial mid-zone fatigue accumulation. Progressive temporal worsening is inconsistent with purely reversible effects. Probable blistering-type intermediary stage preceding delamination [18].	Outerbridge Grade II; ICRS Grade IIA–IIB; OARSI Grade 2.
Severe degradation	Pronounced blushing with high D/PTR, visible delamination, and permanent plastic deformation persisting after 14-h PBS hydration recovery.	Irreversible structural failure confirmed by failure to recover after extended hydration. Indicates collagen network disruption beyond transient fluid-flow mechanisms [19,20]. Delamination confirms middle-zone shear fatigue failure [21,22].	Outerbridge Grade III–IV; ICRS Grade III; OARSI Grade 3–4.

TD: Tissue Degradation. D/PTR: Degraded/Pre-Test Ratio. PBS, phosphate-buffered saline. Outerbridge scale adapted from Pallan (2016) [14], visual damage grading from Dubey (2023) [17].

**Table 3:** Mean Hertzian contact values for the hemiarthroplasty joints (SD).

Group	RC	Femoral Head Diameter [mm]	Contact Pressure [MPa]
Natural Tissue	0	40.75 (2.25)	2.02 (0.07)
Negative	−0.75	38.5 (0.0)	3.32 (0.02)
Small	0.19	40.4 (0.25)	3.3 (0.01)
Large	1.88	39 (1.63)	3.49 (0.07)
Extra Large	3.38	41 (1.08)	3.47 (0.06)

## Data Availability

Data is available through the University of Leeds Library website, a DOI will be minted upon acceptance, references are already in place within the document.

## Abbreviations

The following abbreviations are used in this manuscript:

HA	Hemiarthroplasty
THA	Total Hip Replacement
RC	Radial Clearance
TAL	Transversal Acetabular Ligament
COR	Centre of Rotation
PBS	Phosphate-Buffered Saline
D/PTR	Degraded/Pre-Test Ratio
IQR	Interquartile Range
AHS	Anatomical Hip Simulator
